# Calculation of Thermal Expansion Coefficient of Rare Earth Zirconate System at High Temperature by First Principles

**DOI:** 10.3390/ma15062264

**Published:** 2022-03-18

**Authors:** Xingqi Wang, Xue Bai, Wei Xiao, Yuyang Liu, Xiaoning Li, Jianwei Wang, Cheng Peng, Lijun Wang, Xingming Wang

**Affiliations:** 1 National Engineering Research Center of Environment-Friendly Metallurgy in Producing Premium Non-ferrous Metals, GRINM Group Co., Ltd., Beijing 101407, China; target_material@foxmail.com (X.W.); baixue1986@163.com (X.B.); liuyuyangy@126.com (Y.L.); lixn0305@foxmail.com (X.L.); pc030319@126.com (C.P.); gold@grinm.com (L.W.); 2 GRINM Resources and Environment Tech. Co., Ltd., Beijing 101407, China; 3 General Research Institute for Nonferrous Metals, Beijing 100088, China; xiaowei@grinm.com (W.X.); wangjianwei@grinm.com (J.W.); 4 GRIMAT Engineering Institute Co., Ltd., Beijing 101407, China

**Keywords:** thermal expansion coefficient, rare earth zirconates, first principles calculation, Yb doped Gd_2_Zr_2_O_7_

## Abstract

Compounds of rare earth zirconates with pyrochlore structure are candidates for the application of thermal barrier coatings of next generation. In order to modify the mechanic properties and maintain the low thermal conductivity, other trivalent rare-earth element substitution is commonly used. Presently, investigation on the evaluation of the property of thermal expansion is attracting more attention. In this paper, a feature parameter of thermal expansion coefficient at high temperature (α_∞_) was proposed by combining Grüneisen’s equation and the Debye heat capacity model. Using α_∞_ model, the thermal expansion property of different compounds can be easily figured out by first principles. Firstly, α_∞_ of ZrO_2_, HfO_2_, were calculated, and results are in good agreement with the experimental data from the literature. Moreover, α_∞_ of La_2_Zr_2_O_7_, Pr_2_Zr_2_O_7_, Gd_2_Zr_2_O_7_, and Dy_2_Zr_2_O_7_ were calculated, and results demonstrated that the model of α_∞_ is a useful tool to predict the thermal expansion coefficient at high temperature. Finally, Gd_2_Zr_2_O_7_ with 4 different Yb dopant concentrations (Gd_1__-x_Yb_x_)_2_Zr_2_O_7_ (x = 0, 0.125, 0.3125, 0.5) were calculated. Comparing with the experimental data from the literature, the calculation results showed the same tendency with the increasing of Yb concentration.

## 1. Introduction

It is well known that yttria-stabilized zirconia with a mass fraction of 8%(8YSZ) is widely used as top coating of thermal barrier coatings (TBCs) for aero engines [1,2]. However, with the continuously increasing demand of the thrust-to-weight ratio, 8YSZ is not workable because of its phase change and sintering since the temperature of the turbine front inlet is much greater than 1200 °C [3,4]. Gd_2_Zr_2_O_7_, as the representative of rare earth zirconates, is evidently one of the most promising candidates for the application of next generation TBCs due to its lower thermal conductivity and higher phase stability [5,6]. However, it still suffers from the problem that mechanical properties are not high enough and thermal cycling performance is poor [7]. The thermal expansion properties play the key role. Comparatively, the thermal expansion coefficient of rare earth zirconates is about 9–10 × 10^−6^ K^−1^(1073 K) [8], which is much lower than that of 8YSZ, about 11 × 10^−6^ K^−1^(1073 K) [3]. The thermal expansion coefficient of NiCoCrAlY bonding layer, is about 17.5 × 10^−6^ K^−1^(1273 K) [5]. The mismatch of the thermal expansion between the ceramic top layer and the bonding layer causes thermal stresses during thermal cycling, which can lead to cracks and failure of the TBCs system [9,10].

In order to improve the thermal expansion property of rare earth zirconates, doping with other trivalent rare earth elements is typically used. Excellent thermophysical properties such as high thermal stability, lower thermal conductivity, and high thermal expansion have been demonstrated in doped rare earth zirconates, such as Sm_2_(Zr_1-x_Ce_x_)_2_O_7_, (Nd_1-x_Gd_x_)_2_Zr_2_O_7_, Gd_2_(Zr_1-x_Ti_x_)_2_O_7_ and (Sm_1-x_Gd_x_)Zr_2_O_7_ [6,11,12,13], etc.

Nevertheless, the research described above was carried out mainly by experiments, which are time costly and not fully adequate. For instance, heat conduction and thermal expansion of materials are closely related to lattice vibrations. However, to investigate the lattice vibrations of materials experimentally, neutron scattering or Raman spectroscopy is required [14]. Correspondingly, computational material simulations are more efficiently implemented to accelerate the design of materials. As we know, the first principles exhibit powerful capabilities in material design. Zhao [15] investigated the structure, mechanical properties, minimum thermal conductivity, and electronic properties of a series of Gd-site and Zr-site substituted Gd_2_Zr_2_O_7_ pyrochlores by first principles. Atsushi Togo [16] calculated the thermal expansion properties of Ti_3_SiC_2_, Ti_3_AlC_2_, and Ti_3_GeC_2_ by the first principles combing with quasi-harmonic approximation (QHA). Feng [17] investigated the thermal expansion properties of rare earth zirconates (Ln_2_Zr_2_O_7_, Ln = La, Nd, Sm and Gd) of pyrochlore structures by using first principles. Meanwhile, the calculation of properties of doped compounds by first principles is very difficult especially for the calculation of the thermal expansion property. For instance, the conventional cell of Gd_2_Zr_2_O_7_ contains 88 atoms, and cell expansion must be performed to obtain solid solution structures with different doping concentrations, which makes the calculation of phonons extremely difficult.

Classically, Grüneisen proposed a thermal expansion equation [18], in which the thermal expansion coefficient(α) is determined by elastic properties and heat capacity (*C_V_*). Further, *C_V_* is the function of Debye temperature and temperature. When the temperature is much greater than Debye temperature, *C_V_* can be considered as a constant. In this case, the calculation of α is simplified to the calculation of elastic properties, which makes the calculation much easier and faster. Coincidentally, the TBCs for aero engines operate at a high temperature, which is much higher than Debye temperature. Moreover, investigations showed that the coefficient of thermal expansion gradually increases with temperature increasing at high temperature, but the increasing rate gradually decreases. Reasonably, the thermal expansion coefficient at super high temperature(α_∞_) can be used as a comparison factor to characterize the thermal expansion property of different dopant/concentration compound materials.

Therefore, in this paper, based on the Grüneisen’s equation, a computational model of α_∞_ was developed, by which the calculation of a series of rare earth zirconates were implemented by the first principles.

## 2. Methodology

The supercell containing 88 atoms of Gd_2_Zr_2_O_7_ was used for calculation. The structures of Yb doped Gd_2_Zr_2_O_7_ were formed by replacing Gd atoms with different amounts of Yb atoms. The structural models for (Gd_1-x_Yb_x_)_2_Zr_2_O_7_ were built using the cluster expansion approach by calculating the lowest forming energy [19,20,21]. Further, the structures were optimized by the Birch–Murnaghan equation of state [22]. The elastic constants of the material were calculated by the stress–strain method [23].

The first principles calculations were based on density functional theory using the Vienna Ab initio Simulation Package (VASP) [24] with the generalized gradient approximation (GGA) for exchange-correlation energy, in the form of Perdew–Burke–Ernzerhof (PBE) [25]. The kinetic cut-off energy for the plane wave expansion was taken to be 600 eV in the Brillouin zone integrations using 2 × 2 × 2 k points. The average force acting on ions was reduced to 0.05 eV/Ang. Valence electrons included for distinct atoms were O 2s^2^2p^4^, Zr 5s^1^4d^3^, Hf 5d^3^6s^1^, Gd 6s^2^5p^6^5d^1^, Yb 6s^2^5p^6^.

## 3. Results

### 3.1. Yb Doped Gd_2_Zr_2_O_7_ Structure

**Figure 1:**
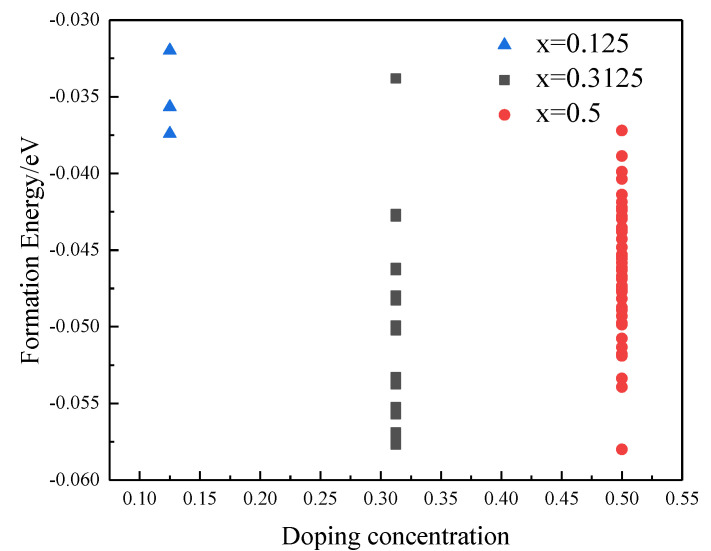
Calculation of E of (Gd_1__-x_Yb_x_)_2_Zr_2_O_7_ (x = 0.125, 0.3125, 0.5).

**Figure 2:**
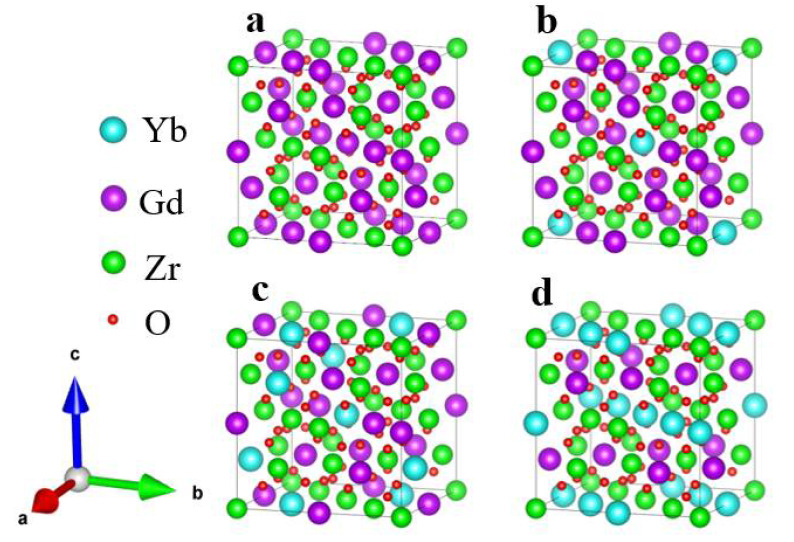
Geometrical configurations of (Gd_1__-x_Yb_x_)_2_Zr_2_O_7_, (**a**) Gd_2_Zr_2_O_7_; (**b**) (Gd_0.875_Yb_0.125_)_2_Zr_2_O_7_; (**c**) (Gd_0.6875_Yb_0.3125_)_2_Zr_2_O_7_; (**d**) (Gd_0.5_Yb_0.5_)_2_Zr_2_O_7_.

The supercell of Gd_2_Zr_2_O_7_ contains 88 atoms, including 16 Gd atoms, 16 Zr atoms and 56 oxygen atoms. A total of 2, 5 and 8 Yb atoms were used to replace the Gd atoms to obtain three different concentrations: (Gd_0.875_Yb_0.125_)_2_Zr_2_O_7_, (Gd_0.875_Yb_0.3125_)_2_Zr_2_O_7_, and (Gd_0.5_Yb_0.5_)_2_Zr_2_O_7_, respectively. Correspondingly, the possible numbers of (Gd_1-x_Yb_x_)_2_Zr_2_O_7_ doped structures were $C_{16}^{2}$, $C_{16}^{5}$, and $C_{16}^{8}$. Excluding the equivalent structures, the number of unequal possible doped structures are 3, 35, and 97, separately. The forming energy *E* of each structure was calculated using the cluster expansion approach, according to Equation (1) [21], and the final doped structure was identified by the structure with the lowest forming energy.

(1) $$E=\frac{\left(E_{0}-n_{1}\times E_{1}-n_{2}\times E_{2}\right)}{\left(n_{1}+n_{2}\right)}$$

wherein *E*_0_ is the energy of the doped structure; *E*_1_ and *E*_2_ are the energy of the single cell of Gd_2_Zr_2_O_7_ and Yb_2_Zr_2_O_7_, and n_1_ and n_2_ are the number of dopant atoms, respectively. The calculation results were shown in Figure 1. According to the lowest forming energy, the geometrical configurations of three different doping structures were elaborated in Figure 2.

### 3.2. Lattice Constant and Elastic Modulus

Based on the geometrical configurations shown in Figure 2, the lattice constants and elastic modulus were calculated and listed in Table 1.

It can be seen that the lattice constants decrease with the increasing of Yb dopant. The calculated bulk and shear modulus of Gd_2_Zr_2_O_7_ are 176.6 Gpa and 91.9 GPa, respectively, which meets agreement with the data measured by experiments [26]. Generally, the bulk modulus and shear modulus of (Gd_1__-x_Yb_x_)_2_Zr_2_O_7_ decrease with the increasing of Yb content, which are possibly caused by structure change. Subramanian M A [27] pointed out that doping of Yb atoms reduce the average cation radius ratio r(A^3+^)/r(B^4+^) and change the crystal structure of Gd_2_Zr_2_O_7_ from pyrochlore to disorders in the structures. For the cubic phase, there are three independent elastic constants, C_11_, C_12_, and C_44_ [28]. The calculated elastic constants are all positive, satisfying the generalized elastic stability criterion, namely, C_11_ + 2C_12_ > 0; C_44_ > 0; C_11_ − C_12_ > 0, indicating that all studied structures are mechanically stable [29]. According to Pugh’s theory [30], when *G*/*B* < 0.5, the material is ductile; otherwise, the material is brittle. The *G*/*B* value of all materials calculated is greater than 0.5, indicating that they are brittle materials.

### 3.3. Thermal Expansion of Rare Earth Zirconates System

According to Grüneisen’s equation [18], the volumetric coefficient of thermal expansion (*β*) can be expressed as Equation (2).

(2) $$\beta =\frac{\gamma }{B}\frac{C_{V}}{V}$$

Here, *γ* is Grüneisen’s constant, *C_V_* is the heat capacity, *B* is the bulk modulus, and *V* is the molar volume.

As we know, the rare earth zirconate is in cubic phase. For cubic phase, *γ* is defined as function of the Poisson’s ratio (*μ*) as the following Equation (3) [31].

(3) $$\gamma =\frac{3}{2}\left(\frac{1+\mu }{2-3\mu }\right)$$

*C_V_* can be calculated by the Debye heat capacity model as the following Equation (4) [32]:

(4) $$C_{V}=9Nk_{B}{\left(\frac{T}{T_{D}}\right)}^{3}\int_{0}^{\frac{T_{D}}{T}}\frac{x^{4}e^{x}}{{\left(e^{x}-1\right)}^{2}}dx$$

wherein *N* = *nN_A_*, *N_A_* is Avogadro’s constant, and *n* is the number of atoms in the molecular formula; *k_B_* is the Boltzmann constant; *T_D_* is the Debye temperature. Meanwhile, *T_D_* can be calculated by the following Equation (5) [33].

(5) $$T_{D}=\frac{h}{k_{B}}{\left[\frac{3n}{4\pi }\left(\frac{N_{A}\rho }{M}\right)\right]}^{1/3}\upsilon_{m}$$

wherein *h*, *ρ*, *M*, *υ_m_* is Planck’s constant, theoretical density, relative molecular mass, and speed of sound, respectively. *υ_m_* is defined as Equation (6).

(6) $$\upsilon_{m}=\sqrt[{3}]{3}{\left(\frac{1}{\upsilon_{L}^{3}}+\frac{2}{\upsilon_{S}^{3}}\right)}^{-1/3}$$

*υ_L_*, *υ_S_* is the longitudinal and transverse sound velocity and can be expressed as Equations (7) and (8), separately.

(7) $$\upsilon_{L}=\sqrt{\frac{3B+4G}{3\rho }}$$

(8) $$\upsilon_{S}=\sqrt{\frac{G}{\rho }}$$

For cubic crystals, *β* = 3*α*, *α* is the linear expansion coefficient [17]. From the above Equations (4)–(8), it can be deduced that α can be expressed as Equation (9):

(9) $$\alpha =\frac{9Nk_{B}\frac{1+\mu }{2-3\mu }{\left(\frac{T}{T_{D}}\right)}^{3}\int_{0}^{\frac{T_{D}}{T}}\frac{x^{4}e^{x}}{{\left(e^{x}-1\right)}^{2}}dx}{2BV}$$

Moreover, Equation (9) can be rewritten as Equation (10).

(10) $$\alpha =\left(\frac{9Nk_{B}\frac{1+\mu }{2-3\mu }T^{3}\int_{0}^{\frac{T_{D}}{T}}\frac{x^{4}e^{x}}{{\left(e^{x}-1\right)}^{2}}dx}{2BV}\right)T_{D}^{-3}$$

According to the calculation result shown in Table 1, *B*, *μ*, and *V* of different (Gd_1__-x_Yb_x_)_2_Zr_2_O_7_ vary within 4% difference, which can be considered as constant. Thus, it is indicated from Equation (10) that α is proportional to *T_D_^−3^* at the same temperature, which is compliant with Ruffa’s equation [34].

Furthermore, supposing that the temperature is much greater than the Debye temperature, the integral term in Equation (9) can be mathematically simplified and finally Equation (11) can be obtained.

(11) $$\alpha_{\infty }=\frac{3Nk_{B}\frac{1+\mu }{2-3\mu }}{2BV}$$

Here, α_∞_ representatives the linear thermal expansion coefficient at super high temperature. Actually, the TBCs are working under the temperature (e.g., the temperature of combustion chamber in F135 turbine engine can be up to 2253 K [35]) much higher than *T_D_* of rare earth zirconate (about 500 K) [36]. Another one, α of rare earth zirconate, increases with the increase in temperature, meanwhile the increasing rate slows down more and more [37,38,39]. Therefore, α_∞_ can be likely used to compare the difference of thermal expansion property of different dopant/concentration for the same compound.

### 3.4. The Validity of α_∞_ Model

Cubic ZrO_2_ and HfO_2_ are of typical fluorite structure which is the same as that of rare earth zirconates [40]. Firstly, the lattice parameter and elastic properties were calculated by first principles. Secondly utilizing the α_∞_ model, the thermal expansion properties of cubic ZrO_2_ and HfO_2_ were calculated, and both results were listed in Table 2. Comparing to the data from the material project database, calculation results of lattice parameter and elastic properties are very close to the same level. Hong [41] and Irshad, K.A. [42] measured the linear thermal expansion coefficient (α) of cubic ZrO_2_ and HfO_2_ by in situ high-temperature X-ray diffraction, and it is (12 ± 3) × 10^−^^6^ K^−^^1^ and 8.80 × 10^−6^ K^−^^1^, respectively. Comparably, the calculated results of α_∞_ are 9.72 × 10^−^^6^ K^−^^1^ and 9.05 × 10^−6^ K^−^^1^. It is revealed that both are a good match.

**Figure 3:**
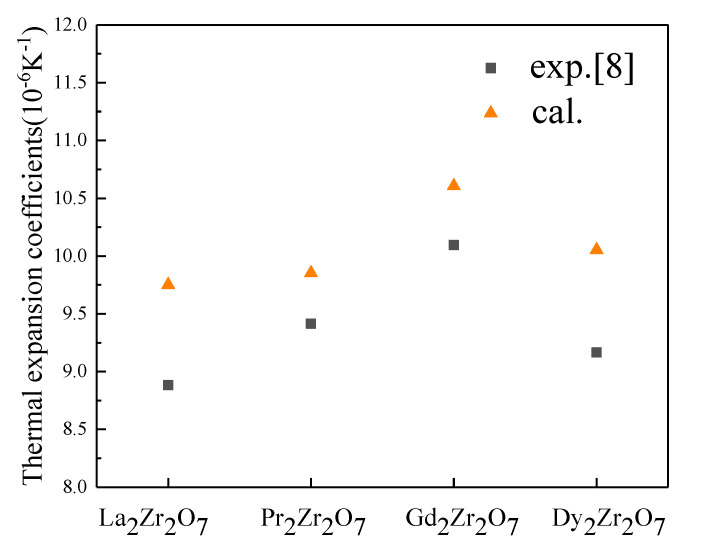
α_∞_ and α of La_2_Zr_2_O_7_, Pr_2_Zr_2_O_7_, Gd_2_Zr_2_O_7_, and Dy_2_Zr_2_O_7_.

In order to further verify the validity of the α_∞_ model, α_∞_ of serial rare earth zirconates, including La_2_Zr_2_O_7_, Pr_2_Zr_2_O_7_, Gd_2_Zr_2_O_7_, and Dy_2_Zr_2_O_7_ were calculated. The data of elastic property was cited from the literature [36], and both were shown in Table 3. α_∞_ and α were plotted in Figure 3. Compared to α measured by experiment [8], α_∞_ are clearly higher because α were measured at 800 °C which is not too much higher than Debye temperature. Henry Lehmann [37] measured the thermal expansion coefficients of Gd_2_Zr_2_O_7_ and La_2_Zr_2_O_7_, which were 10.652 × 10^−6^ K^−1^ (1473 K) and 9.09 × 10^−6^ K^−1^ (1373 K), respectively. The results are very close to α_∞_ calculated. It is further demonstrated that α_∞_ can be a useful tool to predict the thermal expansion coefficient at high temperature.

### 3.5. The Effect of Yb Doping of Gd_2_Zr_2_O_7_ on α_∞_

α_∞_ of Gd_2_Zr_2_O_7_ with 4 different Yb doping contents Gd_2_Zr_2_O_7_, (Gd_0.875_Yb_0.125_)_2_Zr_2_O_7_, (Gd_0.875_Yb_0.3125_)_2_Zr_2_O_7_, and (Gd_0.5_Yb_0.5_)_2_Zr_2_O_7_ were calculated. Table 4 shows the calculation result of theoretical density, sound velocity, and Debye temperature.

It can be seen that *T_D_* of Gd_2_Zr_2_O_7_ was calculated to be 511 K, which is in good agreement with that measured by Toshiaki Kawano [8]. *T_D_* is decreases with the increase of Yb dopant, which is dominated by the decrease of average velocity of sound (*υ_m_*).

**Figure 4:**
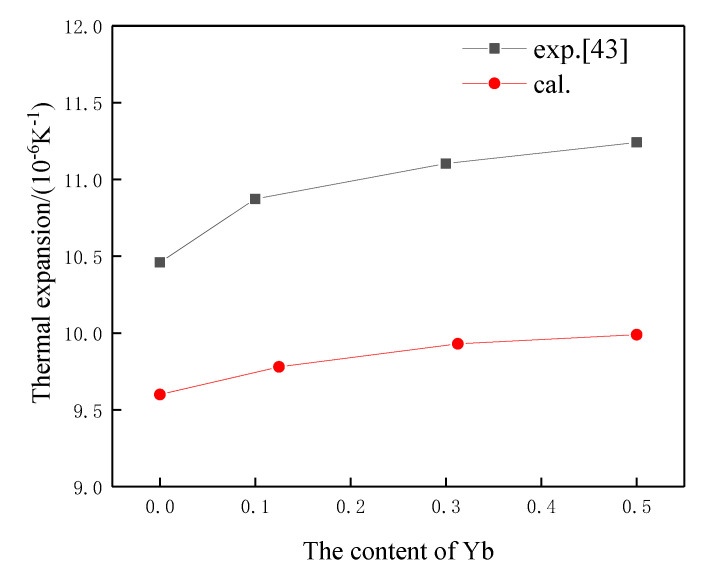
α_∞_ and α of (Gd_1__-x_Yb_x_)_2_Zr_2_O_7_.

Figure 4 shows the difference between calculated α_∞_ and α measured by experiment at 1073 K [43].

It is clear that Yb doping can increase the thermal coefficient greatly because theoretical density increases meanwhile *T_D_* decreases with Yb doping, as shown in Table 4. However, α_∞_ is a bit lower than α. Feng [17] also discussed and explained the problem. Actually, the first principles were developed based on the material being from an ideally perfect crystal. However, for real bulk materials, various defects (e.g., vacancies and dislocations) and pores ineluctably existed. In general, the total energy of a crystal with defects is higher than that of an ideally perfect crystal, and the anharmonic effect may be affected by various defects and pores in the structure. In addition, the density of the tested ceramic coupon is certainly lower than that of theoretical density. That is the reason why the thermal expansion coefficient measured by experiment is higher than that calculated.

Figure 4 also reveals that both α_∞_ and α increase with an increase in Yb content. The increase of thermal coefficient is more remarkable with lower Yb doping concentration. With higher Yb doping concentration, the growth rate slows down. Both the measured and calculated results have the same tendency.

**Figure 5:**
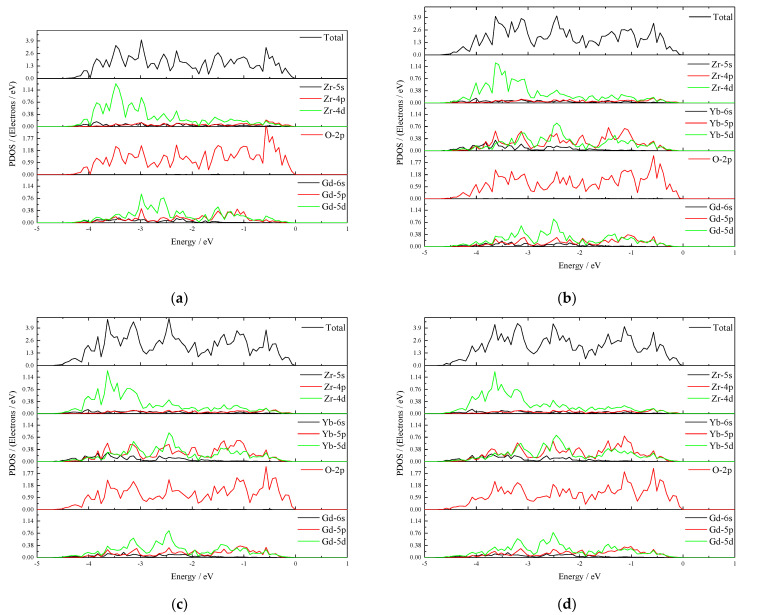
Partial density of states of (Gd_1__-x_Yb_x_)_2_Zr_2_O_7_: (**a**) x = 0; (**b**) x = 0.125; (**c**) x = 0.3125; (**d**) x = 0.5.

Thermal expansion is related to the crystal structure and the electronic structure [44]. The PDOS of (Gd_1__-x_Yb_x_)_2_Zr_2_O_7_ crystals are shown in Figure 5. In terms of bonding, Gd/Yb-5d, Zr-4d, and O-2p overlap, which means that the electronic state around the Fermi level is primarily determined by the relatively weak p-d bond between O-2p and Zr-4d (or Gd/Yb-5d). The main change from doping is the relative position changes in Gd/Yb- 5d, which may be caused by the difference in the valence electron layers between Gd and Yb. With the increase of Yb content, the total state density curve moves slightly to the lower energy level. Low crystal energy means high coefficient of thermal expansion [45], and the rare earth element Yb influences the O-2p states due to hybridization [44]. The p-d bond strength decreased with the increase in Yb content. According to the value of the PDOS ordinate, the PDOS of Zr-4d is the largest, so the p-d bond strength of Zr-O is higher than Yb-O and Gd-O.

## 4. Conclusions

Rare earth zirconates are candidates for next generation TBCs, and it is important to develop a method to characterize the thermal expansion property. Combining the Grüneisen’s equation and the Debye heat capacity model, an efficient model of α_∞_ to characterize the thermal expansion coefficient at super high temperature was established. Firstly, using the α_∞_ model, the high temperature thermal expansion coefficients of cubic ZrO_2_ and cubic HfO_2_ were calculated to be 9.72 × 10^−6^ K^−1^and 9.05 × 10^−6^ K^−1^, respectively, which are in agreement with those shown in the literature. Secondly, α_∞_ of serial rare earth zirconates, including La_2_Zr_2_O_7_, Pr_2_Zr_2_O_7_, Gd_2_Zr_2_O_7_, and Dy_2_Zr_2_O_7_ were calculated, and results demonstrated that α_∞_ can be a useful tool to predict the thermal expansion coefficient at high temperature. Lastly, α_∞_ of (Gd_1__-x_Yb_x_)_2_Zr_2_O_7_ with four different doping contents were calculated, and results showed the same tendency as that measured by experiments. Generally, by characterizing the thermal expansion coefficient at high temperature through the elastic properties and Debye temperature of the material, the complicated calculation of phonon spectrum can be avoided. Thus, the model of α_∞_ has the broad application prospect to predict the thermal expansion property at high temperature for other rare earth zirconates.

## Figures and Tables

**Table 1:** Lattice constant, the elastic constants (C_11_, C_12_, and C_44_), bulk modulus (*B*), shear modulus (*G*), and Poisson’s ratio (*μ*) of rare earth zirconates.

	a_0_/(nm)	C_11_/(GPa)	C_12_/(GPa)	C_44_/(GPa)	*B*/(GPa)	*G*/(GPa)	*G*/*B*	*μ*
Gd_2_Zr_2_O_7_, cal.	1.056	316.4	106.7	84.2	176.6	91.9	0.52	0.278
Gd_2_Zr_2_O_7_, exp. [26]	1.054				174	93		
(Gd_0.875_Yb_0.125_)_2_Zr_2_O_7_	1.055	312.3	100.2	83.4	170.6	91.7	0.54	0.272
(Gd_0.6875_Yb_0.3125_)_2_Zr_2_O_7_	1.052	308.4	96.7	83	167.2	91.6	0.55	0.269
(Gd_0.5_Yb_0.5_)_2_Zr_2_O_7_	1.050	310.4	96	82.5	167.7	91.7	0.55	0.269

**Table 2:** Lattice constant, bulk modulus (*B*), shear modulus (*G*), Poisson’s ratio (*μ*), and α_∞_ of ZrO_2_ and HfO_2_.

	a_0_/(nm)	*B*/(GPa)	*G*/(GPa)	*μ*	α_∞_ (K^−1^)
ZrO_2_, cal.	0.512	238.5	100.6	0.316	9.72 × 10^−6^
ZrO_2_ [a]	0.515	235	103	0.31	
HfO_2_, cal.	0.508	253.8	112.6	0.307	9.05 × 10^−6^
HfO_2_ [b]	0.508	248	115	0.3	

[a] Materials data on ZrO_2_ (SG:225) by Materials Project. ID:mp-1565. [b] Materials data on HfO_2_ (SG:225) by Materials Project. ID:mp-550893.

**Table 3:** Lattice constant a_0_, bulk modulus (*B*), shear modulus (*G*), Poisson’s ratio (*μ*) of rare earth zirconates system [36].

	a_0_/(nm)	*B*/(GPa)	*G*/(GPa)	*μ*	Thermal Expansion Coefficient/(10^−6^K^−1^)	
					α_∞_	α/(1073 K) [8]
La_2_Zr_2_O_7_	1.081	176	87	0.302	9.755	8.883
Pr_2_Zr_2_O_7_	1.072	155	103	0.26	9.857	9.415
Gd_2_Zr_2_O_7_	1.052	165	63	0.284	10.61	10.094
Dy_2_Zr_2_O_7_	1.054	164	90	0.268	10.057	9.166

**Table 4:** Density (ρ), longitudinal wave velocity (*υ_L_*), shear wave velocity of sound (*υ_S_*), average velocity of sound (*υ_m_*) and Debye temperature (*T_D_*) of rare earth zirconates.

	*ρ*/(kg·m^−3^)	*υ_L_*/(m·s^−1^)	*υ_S_*/(m·s^−1^)	*υ_m_*/(m·s^−1^)	*T_D_*/(K)
Gd_2_Zr_2_O_7_	6868	6600	3659	4075	511
(Gd_0.875_Yb_0.125_)_2_Zr_2_O_7_	6944	6496	3635	4046	508
(Gd_0.6875_Yb_0.3125_)_2_Zr_2_O_7_	7059	6402	3602	4007	504
(Gd_0.5_Yb_0.5_)_2_Zr_2_O_7_	7176	6357	3574	3977	502

## Data Availability

The data presented in this study are available upon request from the corresponding author.
